# Lower Incidence of Hepatobiliary Cancer in *Helicobacter pylori-*Infected Persons: A Cohort Study of 53.633 Persons

**DOI:** 10.1016/j.jceh.2021.11.013

**Published:** 2021-11-26

**Authors:** Linda S. Kornerup, Peter Jepsen, Lars E. Bartels, Jens F. Dahlerup, Hendrik Vilstrup

**Affiliations:** ∗ Department of Hepatology and Gastroenterology, Aarhus University Hospital, Aarhus, Denmark; † Department of Clinical Epidemiology, Aarhus University Hospital, Aarhus, Denmark; ‡ Department of Rheumatology, Aarhus University Hospital, Aarhus, Denmark

**Keywords:** *Helicobacter pylori*, liver cancer, hepatocellular carcinoma, cholangiocarcinoma, gastric cancer, HP, *Helicobacter pylori*, MALT, mucosa-associated lymphoid tissue, HCC, hepatocellular carcinoma, UBT, urea breath test, CPR, Central Personal Identification Registry Number, ICD, International Classification of Diseases

## Abstract

**Background and aims:**

*Helicobacter pylori* (HP) is known to be involved in intestinal carcinogenesis. As regards hepatobiliary cancers, there are few and inconsistent reports. We investigated HP infection and its association with the incidence of hepatobiliary cancers in a large cohort study. The cohort's appropriateness for the purpose was gauged by its ability to identify the established risk relation to gastric cancer.

**Methods:**

This historical study was performed in the Central Denmark Region. Patients were included from primary healthcare after being tested for HP infection with a urea breath test. Patients’ diagnoses, age, gender, and country of birth were obtained from Danish national administrative registries. Cox regression was used to compare incidences of hepatobiliary and gastric cancer between HP-positive and HP-negative persons, adjusting for confounding variables.

**Results:**

A total of 53,633 persons were included and 10,553 were tested HP-positive. They were followed for a median of 4.6 years (total 250,515 person-years). We found 64 hepatobiliary cancers, with a markedly lower incidence in HP-positive persons; HR = 0.27 (95% CI 0.11–0.68). A higher incidence of gastric cancer in HP-positive persons was confirmed (HR = 1.99 (95% CI 1.35–2.94)).

**Conclusion:**

The incidence of hepatobiliary cancers was remarkably lower in HP-infected persons after adjusting for age, gender, cirrhosis, alcohol-related diagnoses, chronic viral hepatitis, and country of origin. We found no methodological cause for this unexpected finding, and the pathogenic links between the infection and cancer remain to be identified. Our results must be confirmed in a similar cohort.

*Helicobacter pylori* (HP) is a gram-negative bacterium colonising the gastric mucosa. HP infection is very common worldwide, and the prevalence increases with age.^1^, ^2^, ^3^, ^4^ The bacterium has been classified as a carcinogen and increases the risks of gastric cancer and mucosa-associated lymphoid tissue lymphoma.^5^, ^6^, ^7^ Some indications exist showing that HP may reduce the risk of oesophagus cancer,^8^^,^^9^ and it has been suggested that the risk may increase after HP eradication.

Hepatobiliary cancer covers primary cancers of the liver, the biliary tree, and the gallbladder. Primary liver cancer is the fifth most common cancer and one of the leading causes of cancer-related death worldwide.^10^ Hepatocellular carcinoma (HCC) comprises 90% of all primary liver cancers and develops predominantly in patients with underlying chronic liver disease.^11^^,^^12^

Any association between such prevalent diseases is of obvious pathophysiological and public health interest. Recently, as well a higher prevalence and a lower prevalence of HP DNA in liver tissue with HCC and chronic liver disease are reported, and the findings are of unclear significance.^13^, ^14^, ^15^, ^16^, ^17^, ^18^, ^19^, ^20^ A single study from Ethiopia describes a higher prevalence of HP antigen in faeces in HCC patients compared with controls.^21^ These studies are cross-sectional and deal with the association between HP and hepatobiliary cancer in patients with already known liver disease. No evidence exists of hepatic HP infection or colonisation. A Finnish nested case–control study from 2014 found that seropositivity to *H. pylori* proteins was associated with an increased risk of biliary tract cancers.^22^ However, the study size was small, and the cohort was based on a previous study of lung cancer in male smokers, and thus, the patients were highly selected. This was also displayed by the rate of seropositivity as high as 88–100%. An Australian case–control study and meta-analysis investigated pancreatic cancer risk reported a tendency towards lower cancer risk in individuals seropositive for the *H. pylori* protein, CagA, and a higher risk in seronegative individuals.^23^

HP is a recognised carcinogen of the gastrointestinal tract, and it remains unsettled if and how HP relates to hepatobiliary cancer. We had access to a dataset of people tested for the presence or absence of HP with long-term follow-up. On this background, we investigated the association of HP infection and the incidence of hepatobiliary cancer in a population-based cohort.

## Materials and methods

The study was approved by the National Board of Health and by the Danish Data Protection Agency (journal number 1-16-02-145-14).

According to Danish law, approval from the Danish Committee on Health Research Ethics was not necessary. As this is a register-based study, written consent from participants was not required.

### Study Design, Participants, and Setting

The current historical cohort study includes a total of 53,633 persons who underwent a urea breath test (UBT) from November 2002 until 31 December 2012, employing a system developed to diagnose and treat HP infections in general practice.^1^ The cohort was established in the eastern part of the Central Denmark Region with a population size of approx. 700.000 persons (of the total Danish population of 5.6 mill.). Generally, a positive UBT leads to the prescription of an HP eradication regime of amoxicillin, chlarithromycin, and a proton pump inhibitor.

### Data Sources and Variables

The test results from the UBT (negative or positive) were extracted from a local laboratory information system. The UBT data were linked to the patients' Danish Central Personal Identification Registry Number (CPR; also coding for gender and age). Via the CPR, the individual records were linked to the Civil Registration System to establish each patient's country of birth and to the National Patient Registry to identify patients with a diagnosis of cancer in the gastrointestinal tract, cirrhosis, chronic hepatitis B and C, and alcohol-related diagnoses (F10.x, excl. F10.0).

The National Patient Registry records dates of hospital admission and discharge, surgical procedures performed, and discharge diagnoses (primary and up to 19 additional) coded by doctors according to the International Classification of Diseases (ICD; the 10th revision was used in this study). The primary ICD code covers the primary symptom or cause of the patient's health system contact. Any additional ICD code covers other diseases of importance for this specific health system contact.

### Outcome

The main outcome was diagnoses of hepatobiliary cancers in the National Patient Registry at the time of UBT and during follow-up. Follow-up ended at diagnosis of hepatobiliary cancer, death, or in censoring on 31 December 2012.

### Statistical Methods

Characteristics of the study population and by HP positivity were tabulated, and data were displayed as median values with interquartile range if relevant (Table 1). Cox regression was used to compare hazard rates of gastrointestinal cancers between HP-positive and HP-negative individuals, adjusting for confounding variables of gender and age. For hepatobiliary cancers, we also adjusted for cirrhosis, alcohol-related diagnoses, chronic hepatitis B and C, and country of origin. Cancers present at the time of UBT were excluded from further statistical analysis.

**Table 1:** Descriptive Statistics of Patients Who Took a First-Time Urea Breath Test (UBT) in General Practice in the Central Denmark Region From 2002 to 2012.

	Total	HP-negative	HP-positive	% Positive
Tests (n)	53,623	43,071	10,552	19.7
Men	21,402	17,008	4394	20.5
Women	32,227	26,069	6158	19.1
Age (years)^a^	42.5 (28.8–56.6)	41.7 (27.9–55.9)	45.1 (32.5–59.5)	
Follow-up (years)	4.6	4.6	4.7	
Born in DK (n)	45,024	39,139	5885	13.1
Born outside DK (n)	8599	3932	4667	54.3
Patients with cirrhosis^b^ (n)	237	165	72	30.4
Patients without cirrhosis^b^ (n)	53,394	42,913	10,481	19.6
Patients with alcohol-related diagnoses^c^ (n)	432	365	67	15.5
Patients without alcohol-related diagnoses^c^ (n)	53,201	42,715	10,486	19.7
Patients with chronic HBV infection^d^	85	45	40	47,1
Patients with chronic HCV infection^e^	125	93	32	25,6
Patients with NAFLD^f^	107	87	20	18,7


DK, Denmark. HBV, hepatitis B virus. HCV, hepatitis C virus. NAFLD, non-alcoholic fatty liver disease.

a Median [25th to 75th percentile].

b ICD10 diagnoses K70.3, K70.4, and K74.6 including ICD10 diagnoses for chronic viral hepatitis B and C; B18.1 and B18.2.

c ICD10 diagnoses F10.x except for F10.0.

d ICD10 diagnoses B18.0 and B18.1.

e ICD10 diagnoses B18.2.

f ICD10 diagnoses K75.8 and K76.0x.

## Results

### Participants and Descriptive Data

The cohort is characterised in Table 1. As particularly noteworthy, a total of 53,633 persons were included, and the prevalence of HP in the entire cohort was 19.7%. The prevalence of HP in individuals born inside Denmark was 13.1%, and it was 54.3% in individuals born outside of Denmark.

Ten persons had a diagnosis of hepatobiliary cancer at the time of UBT (nine HP-negative, one HP-positive) and were excluded.

### Hepatobiliary Cancers

**Figure 1:**
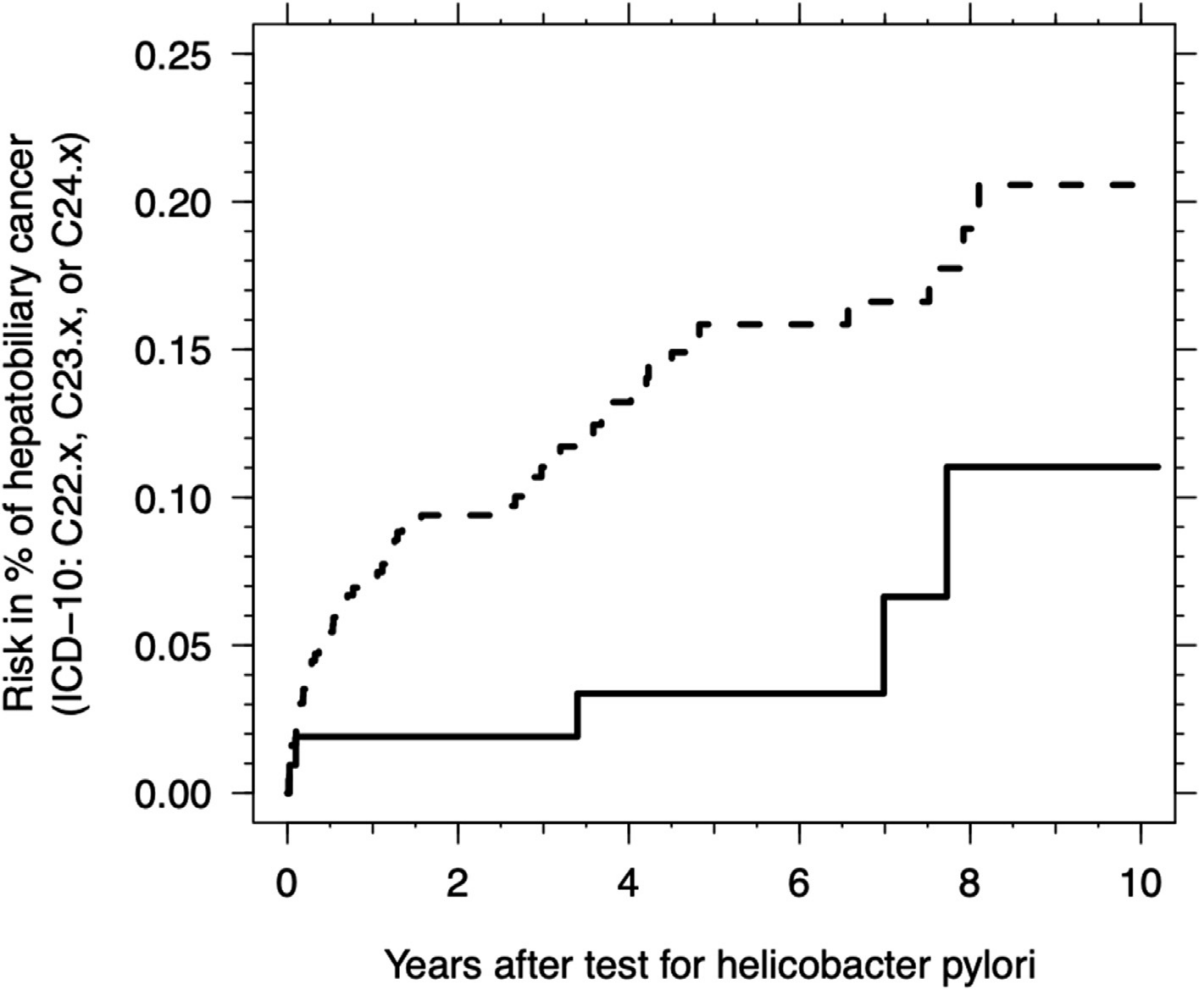
Cumulative risk of hepatobiliary cancer in patients after first-time UBT in HP-negative persons (dashed line) and HP-positive persons (full line).

The median follow-up time was 4.6 years after UBT (total person-years 250,515; 200,149 person-years for HP-negative and 50,366 person-years for HP-positive). During follow-up, a total of 5 HP-positive compared with 59 HP-negative patients were diagnosed with hepatobiliary cancer (see Table 2 for diagnoses and ICD-10 codes). Thus, HP infection was associated with a lower incidence of hepatobiliary cancers. The unadjusted HR was 0.34 (95% CI 0.14–0.85), and adjusting for gender and age strengthened the association to an HR of 0.27 (95% CI 0.11–0.68). Adjusting further for cirrhosis, alcohol-related diagnoses, chronic viral hepatitis, and origin outside of Denmark did not change the association or its precision, with an HR of 0.26 (95% CI 0.10–0.64). Figure 1 displays the cumulative risk of hepatobiliary cancer in HP-positive and HP-negative persons.

**Table 2:** Diagnoses and ICD-10 Codes for Hepatobiliary Cancers Diagnosed During Follow-Up.

Cancer (ICD-10)	HP neg	HP pos	HR	95% CI
All	59	5	0.26	[0.10–0.64]
Hepatocellular carcinoma (C22.0)	20	2	0.30	[0.071–1.31]
Cholangiocarcinoma (C22.1 + C24.x)	20	1	0.16	[0.021–1.16]
Gallbladder (C23)	7	2	0.87	[0.18–4.22]
Malignant neoplasms of liver, unspecified (C22.9)	12	–

HP infection was associated with a higher incidence of gastric cancer (19 cases), yielding an adjusted HR of 1.99 (95% CI 1.35–2.94).

## Discussion

With this large population-based cohort study, we find a markedly lower incidence of hepatobiliary cancer in HP-positive persons compared with HP-negative persons. The association holds true when adjusting for gender, age, alcohol-related diagnoses, cirrhosis, chronic viral hepatitis, and country of birth. This is the first study to date to compare a population defined by HP positivity versus HP negativity for hepatobiliary cancer development.

The role of HP in hepatobiliary cancer has been a hot topic in the last decade, and it is highly debated whether it is a silent bystander or a direct liver carcinogen. Our data, while not contributing clarification of this issue, may be put in perspective by biological findings.

Animal studies show that another Helicobacter species, *Helicobacter hepaticus,* is able to colonise livers of mice and cause hepatitis that may lead to HCC.^24^^,^^25^ A higher prevalence of HP DNA in liver tissue with HCC and chronic liver disease compared with healthy liver tissue is reported, whereas some studies conversely find a lower prevalence.^13^, ^14^, ^15^, ^16^, ^17^, ^18^, ^19^, ^20^ These reports may give rise to the notion that HP could be a risk factor for hepatobiliary cancer. However, it is suggested that viable HP reaches the liver with portal blood by bacterial translocation as a consequence of portal hypertension.^26^ If so, both the presence of HP and the HCC development are mechanistically unrelated consequences of chronic liver disease. It is also suggested that HP DNA in the liver is remnants of phagocytosed bacteria reaching the liver from the stomach with portal blood.^13^ Few studies report culturable HP^27^ and most report only HP DNA by PCR. These reports seem to indicate that there is not longstanding HP infection of the liver, as is the case for the stomach. HP infection predominantly occurs in early childhood and can cause chronic inflammation of the stomach after several decades, and gastric cancer development likely is a consequence of this state. On the other hand, chronic stomach HP inflammation may still relate to a lower hepatobiliary cancer incidence by triggering a protective immune response in the liver by the action of cytokines delivered by portal blood. This may cause a state of enhanced immunologic cancer surveillance. In support, HP causes increased IFN-γ levels, which in the liver can promote anti-cancer immunomodulation.^28^ Importantly, most previous studies are based on populations with HCC or chronic liver disease, whereas our cohort is population-based. In the two Chinese studies,^16^^,^^29^ the prevalence of HBV is very high, while the prevalence of viral hepatitis in Denmark is very low^30^^,^^31^ (HBV 0.24%; HCV 0.21%). However, the prevalence of HP infection in persons with HBV in our study was 47.1%, which is substantially higher than for the total cohort (19.7%). These differences may display separate aspects of pathogenesis. Hence, the Chinese cohorts may not even be comparable with our cohort.

Our study holds several strengths. Healthcare data were available both before and after UBT. The validity of cancer diagnoses including hepatobiliary cancer in The Danish National Patient Registry is very high.^32^, ^33^, ^34^ The sensitivity and specificity of the UBT as employed in this study both exceed 95% in detecting HP^35^ and according to the Maastricht V guideline remain the best-recommended non-invasive tests for detecting HP.^36^ Thus, we believe our study to both provide accurate information on HP status and valid information on the incidence of hepatobiliary cancer in a Danish population. Furthermore, the well-established higher risk of gastric cancer was confirmed in our study, which emphasises the validity of our data.

Nonetheless, our study has limitations. Overall, the total number of cases of hepatobiliary cancers, although not trivial, is small, particularly so for HP-positive persons. Still, to result in non-different HRs, we should have found 14 cancers; that is nine overlooked cases. Considering that such cancers are symptomatic and the tightly woven and free Danish healthcare system, a loss-of-diagnosis of that magnitude is hardly possible. Most diagnoses were HCCs followed by cholangiocarcinoma, and despite the low case numbers resulting from this subdivision, the incidence estimates were similar for both cancers. The same may be said for the very low numbers of gallbladder cancer and other primary liver malignancies (Table 2). This uniform pattern may support that the low incidence among HP-positives was not a chance or spurious finding. However, a causal association might have been more obvious had we found only one cancer type.

HP infection is associated with lower socioeconomic standards, and hepatobiliary cancer is associated with both low and higher socioeconomic standards, depending on the cause of the underlying chronic liver disease. In our cohort, any socioeconomic difference was not expressed as a difference in harmful alcohol use. Anyway, direct adjustment for socioeconomics would have strengthened our analysis.

Possible biases must be addressed as well. Persons with abdominal discomfort and a negative UBT are more likely to be tested for other causes of their symptoms, including hepatobiliary cancer. Another reason could be that persons with undiagnosed hepatobiliary cancer are more likely to be tested for HP, testing negative, and subsequently being diagnosed with hepatobiliary cancer. A bias towards underestimating hepatobiliary cancer in the HP-positive group is theoretically also present. Additional testing may be halted in a person with a positive UBT that might be taken to cause the symptoms. Furthermore, treatment with antibiotics as part of the HP eradication could clear a mild episode of cholangitis as an early sign of biliary cancer. These biases may affect incidence analyses in short term but should practically be abolished by our long follow-up time, as hepatobiliary cancer eventually will cause symptoms and lead to diagnostic work-up. On the other hand, our results could be biased towards the lower prevalence of HP in patients with hepatobiliary cancer, as patients with frequent healthcare contact are more likely to receive antibiotics for several reasons including cancer, which in theory could eradicate possible HP infection. This was examined in a study of HP infection in patients with Crohn's disease and a control group consisting of patients with chronic obstructive lung disease.^37^ A history of antibiotic use did not play a role in the negative association between HP and Crohn's disease. Thus, it should not play a part in our study either.

Overall, the low number of cancer cases and effects of unknown confounders should be kept in mind. However, as Figure 1 shows, the cancer diagnoses were widely distributed throughout the follow-up period. Therefore, our results are not based upon the accumulation of diagnoses within the first year of UBT. There may remain confounders unaccounted for, but such confounders must be very strong to explain our results. Furthermore, to explain our results, such confounders should also be protective for HP, which seems highly unlikely.

We present incidences of cancers occurring after the UBT, and it might be argued that our findings to a degree result from the HP diagnosis and its subsequent eradication. However, the HP exposure time of our positive cohort was about 40 years and the post UBT follow-up 4.6 years and so the post UBT time was likely insignificant – even when being HP eradicated. As previously described for our cohort, HP was successfully eradicated in 82% of HP-positive patients just after the UBT.^38^ This raises the issue of the importance of the short-term eradication medication, but in the same way as above the exposure time factor speaks against any such explanation. Thus, it remains plausible that the results of our study reflect a biological effect of decades of HP infection that lasts after eradication. The prevalence data at UBT were slim but if anything in support.

Our study showed a remarkably lower risk of hepatobiliary cancers in HP-positive persons. We evaluated our results very carefully in search of a methodological cause for the finding and did not find any. As mentioned, our *a priori* expectations were open but if anything, we expected HP infection to be associated with a higher incidence of hepatobiliary cancer, which would have been in better synchrony with the studies reporting a higher prevalence of HP DNA in HCC liver tissue. Our results turned out to refute any such expectations and add a new angle to the discussion of the consequences of HP infection. We hope for confirmation of our results in a similar, suitable cohort. Additionally, animal models could prove helpful in further investigation of the association between *H. pylori* and HCC. An effective mouse model for *H. pylori* infection has been described^39^ and may in combination with a mouse model for chronic liver disease or HCC answer some of the questions raised by our results and those from previous publications.

The incidence of hepatobiliary cancers was lower in HP-infected persons after adjusting for age, gender, alcohol-related diagnoses, cirrhosis, chronic viral hepatitis, and country of origin. This large historical cohort study is the first to suggest that HP infection may play a protective role against hepatobiliary cancer. However, this is an observational study, and our data offer no mechanistic explanation to our findings.

## Credit authorship contribution statement

**Linda S. Kornerup**: Conceptualisation, Methodology, Writing – original draft, Writing – review and editing; **Peter Jepsen**: Conceptualisation, Methodology, Formal analysis, Investigation, Data curation, Writing – review and editing, Supervision; **Lars E. Bartels**: Conceptualisation, Writing – review and editing; **Jens F Dahlerup**: Conceptualisation, Writing – review and editing; **Hendrik Vilstrup**: Conceptualisation, Methodology, Writing – review and editing, Supervision.

## Conflicts of interest

The authors have none to declare.

## Funding

None.
